# Gilbert’s Syndrome and the Gut Microbiota – Insights From the Case-Control BILIHEALTH Study

**DOI:** 10.3389/fcimb.2021.701109

**Published:** 2021-09-16

**Authors:** Patrick A. Zöhrer, Claudia A. Hana, Nazlisadat Seyed Khoei, Christine Mölzer, Marlies Hörmann-Wallner, Anela Tosevska, Daniel Doberer, Rodrig Marculescu, Andrew C. Bulmer, Craig W. Herbold, David Berry, Karl-Heinz Wagner

**Affiliations:** ^1^Department of Nutritional Sciences, Faculty of Life Sciences, University of Vienna, Vienna, Austria; ^2^Research Platform Active Ageing, University of Vienna, Vienna, Austria; ^3^School of Medicine, Medical Sciences and Nutrition, Institute of Medical Sciences, University of Aberdeen, Aberdeen, United Kingdom; ^4^Institute for Dietetics and Nutrition, University of Applied Sciences FH JOANNEUM, Graz, Austria; ^5^Division of Rheumatology, Department of Medicine 3, Medical University of Vienna, Vienna, Austria; ^6^Clinical Institute of Laboratory Medicine, Vienna General Hospital, Medical University of Vienna, Vienna, Austria; ^7^Department of Clinical Pharmacology, Vienna General Hospital, Medical University of Vienna, Vienna, Austria; ^8^School of Pharmacy and Medical Sciences and Menzies Health Institute Queensland, Griffith University, Gold Coast, QLD, Australia; ^9^Division of Microbial Ecology, Department of Microbiology and Ecosystem Science, Centre for Microbiology and Environmental Systems Science, University of Vienna, Vienna, Austria; ^10^Joint Microbiome Facility of the Medical University of Vienna and University of Vienna, Vienna, Austria

**Keywords:** bilirubin, UGT1A1, unconjugated bilirubin, 16S rRNA gene, microbiota, microbiome, colorectal cancer

## Abstract

The heme catabolite bilirubin has anti-inflammatory, anti-oxidative and anti-mutagenic effects and its relation to colorectal cancer (CRC) risk is currently under evaluation. Although the main metabolic steps of bilirubin metabolism, including the formation of stercobilin and urobilin, take place in the human gastrointestinal tract, potential interactions with the human gut microbiota are unexplored. This study investigated, whether gut microbiota composition is altered in Gilbert’s Syndrome (GS), a mild form of chronically elevated serum unconjugated bilirubin (UCB) compared to matched controls. Potential differences in the incidence of CRC-associated bacterial species in GS were also assessed. To this end, a secondary investigation of the BILIHEALTH study was performed, assessing 45 adults with elevated UCB levels (GS) against 45 age- and sex-matched controls (C). Fecal microbiota analysis was performed using 16S rRNA gene sequencing. No association between mildly increased UCB and the composition of the gut microbiota in this healthy cohort was found. The alpha and beta diversity did not differ between C and GS and both groups showed a typical representation of the known dominant phyla. Furthermore, no difference in abundance of *Firmicutes* and *Proteobacteria*, which have been associated with the mucosa of CRC patients were observed between the groups. A sequence related to the *Christensenella minuta* strain YIT 12065 was identified with a weak association value of 0.521 as an indicator species in the GS group. This strain has been previously associated with a lower body mass index, which is typical for the GS phenotype. Overall, sex was the only driver for an identifiable difference in the study groups, as demonstrated by a greater bacterial diversity in women. After adjusting for confounding factors and multiple testing, we can conclude that the GS phenotype does not affect the composition of the human gut microbiota in this generally healthy study group.

## Introduction

Mild hyperbilirubinemia, a benign condition also known as Gilbert´s Syndrome (GS), is usually defined by an unconjugated bilirubin (UCB) blood concentration of above 17.1 µmol/L. The prevalence of GS is remarkably common, affecting 5-10% (depending on ethnicity and sex) of the adult population (Wagner et al., 2018). This condition is influenced by a combination of increased haem catabolism and various underlying promoter polymorphisms in the uridine diphosphoglucuronyltransferase (*UGT1A1*) gene, leading to reduced conjugating activity of this enzyme and therefore, elevated UCB levels. GS is currently assumed to have little or no pathological consequences (Bulmer et al., 2018). A compelling body of evidence has demonstrated that serum bilirubin, a byproduct of hemoglobin breakdown, has substantial anti-inflammatory, anti-oxidative and anti-mutagenic properties (Stocker, 2004; Bulmer et al., 2008; Vitek and Tiribelli, 2020) and that mildly elevated serum bilirubin levels are strongly associated with a reduced prevalence of chronic diseases, such as CVD, Type-2 diabetes and some cancers (Zucker et al., 2004; Wagner et al., 2015; Bulmer et al., 2018; Kwon et al., 2018).

One common link between reduced disease risk and increased UCB concentration is reduced body weight, with consistent reports in the literature demonstrating significantly reduced BMI and occasionally also reduced fat mass in GS when compared to age- and sex-matched controls (Bulmer et al., 2013; Wallner et al., 2013c; Seyed Khoei et al., 2018).

UCB is formed from the breakdown of haem-containing proteins (principally hemoglobin) in the liver/spleen by heme oxygenase, resulting in biliverdin and further enzymatic transformation by biliverdin reductase to bilirubin. Unconjugated bilirubin is removed from the blood by the liver and conjugated by *UGT1A1*. Conjugated bilirubin is then transported to the bowel *via* the bile, where it is enzymatically deconjugated by glucuronidases produced by gut bacteria and then further oxidized and reduced, forming stercobilin and urobilin that can be reabsorbed or excreted in the feces or urine (Wagner et al., 2015; Hamoud et al., 2018).

Since the gut represents a main location of bilirubin metabolism, a link between chronically increased UCB levels and gut health seems likely. We have recently reported on associations between UCB and colorectal cancer (CRC) risk in the European Prospective Investigation into Cancer and nutrition (EPIC) study, whereby serum UCB concentrations were positively associated with CRC risk in men and inversely associated in women (Seyed Khoei et al., 2020).

CRC is the third most common malignancy diagnosed and the fourth leading cause of cancer-related deaths worldwide (Arnold et al., 2017), and is expected to increase by a further 60% over the next decade. This increase is estimated to result in more than 2.2 million additional cases and 1.1 million annual deaths, by the year 2030 (Rawla et al., 2019).

Established CRC risk factors include high consumption of red/processed meat, low intake of dietary fibre, alcohol consumption, smoking, physical inactivity, obesity and height (World Cancer Research Fund/American Institute for Cancer Research, 2018). Increasingly, the gut microbiota has been implicated in CRC. Alterations in gut microbiota composition have been associated with a growing number of diseases, including cancer and particularly CRC (Schwabe and Jobin, 2013). More than 20% of the cancer burden worldwide is attributable to known infectious agents that are often normal residents of the intestinal microbiota (Zur Hausen, 2009).

Although a number of studies (Brennan and Garrett, 2016; Ternes et al., 2020) link certain members of the gut microbiota as causative factors in CRC development, the patho-etiological intricacies are poorly understood (Fong et al., 2020). Several mechanisms, including inflammation, bacterial pathogenicity, genotoxins and oxidative stress have been strongly implicated (Cheng et al., 2020), all of which have potential links to bilirubin metabolism.

Surprisingly, to date, potential associations between (increased) circulating UCB concentrations and the gut microbiota of adults remain unexplored. Therefore, this study aimed to evaluate whether (i) individuals with mildly elevated circulating UCB concentrations (i.e., GS) possess differences in their gut microbiota compared to age- and sex-matched controls, and to determine whether (ii) any observed effects are age- or sex-dependent.

## Materials and Methods

### Participants and Study Design

The “BILIHEALTH” study was designed as an observational case-control study, at a single centre in Vienna, Austria as described more detailed previously (Mölzer et al., 2016; Mölzer et al., 2017).

Briefly, 128 healthy participants between 20 and 80 years of age were recruited from the general Austrian population. During the study, eight were excluded for medical reasons. Exclusion criteria included smoking, excess drinking, routine intake of medication and nutritional supplements, pregnancy, acute and chronic (inflammatory/metabolic) diseases, liver diseases, present or past neoplasia and organ transplants. Each participant completed an initial health check-up which covered fasting blood biochemistry including levels of UCB and liver enzymes, blood pressure, body weight/-height, and questionnaires.

A total of 80 males and 40 females completed the study. This sex distribution is representative of the occurrence of GS in the general population (Wallner et al., 2013a). All participants were age- and sex-matched, and study group allocation (GS vs Control, C) was based on the participants’ fasting serum UCB concentrations (</≥17.1 μM) (Wallner et al., 2013a), as analysed by High-Performance Liquid Chromatography. Most of the GS participants showed visible signs of mild jaundice, observable by a yellowish pigmentation of the skin and the conjunctival membranes over the sclera. Liver parameters and parameters of haemolysis were within the normal ranges. Participants were furthermore allocated to age groups (</≥35 years of age). In order to support diagnosis of GS, all participants of both study groups were required to fast the day before participating in the study, following a 400 kcal fasting protocol (Radu and Atsmon, 2001; Wallner et al., 2013b). Furthermore, a complete overnight fast of 16 ± 1 hour was required before the day of blood sampling.

The study was approved by the Ethics Commission of the Medical University of Vienna (No. 1164/2014), was registered in ClinicalTrials.gov (NCT04792996) and was conducted in accordance with the Declaration of Helsinki. All participants provided signed informed consent prior to study participation.

### Faeces Sampling and Further Exclusion of Subjects

Faecal samples were collected at home by the participants and stored in the refrigerator for not longer than overnight. Samples were handed over in the morning of the screening day at the General Hospital of Vienna, aliquoted to approximately 500 mg in Eppendorf-tubes and stored at -20°C. Eight out of 128 participants were excluded due to exclusion criteria as mentioned above. In addition, 19 participants and their age- and sex-matched controls (in total 30) were excluded due to the lack of faecal sample or undetermined UGT1A1*28-genotype. Consequently, 90 age- and sex-matched participants, were considered for statistical analyses.

### DNA Extraction From Faeces

DNA from faeces samples was extracted using a Phenol/chloroform/isoamyl alcohol extraction protocol as previously described (Griffiths et al., 2000). After bead-beating and centrifugation, DNA was precipitated from the aqueous phase by adding 0.1 volume of 3 M sodium acetate and 0.6 volumes of ice-cold isopropyl alcohol. DNA-pellets were rinsed with 70% ethanol and eluted in 100 µL TlowE-buffer (10 mM Tris-HCl/0.1 mM EDTA dissolved in DEPC-treated water). DNA concentration and quality were determined using a NanoDrop 1000 Spectrometer including ND-1000 operation software set for nucleic acid DNA-50 (Thermo Fisher Scientific). The ratio of the absorbance at 260 and 280 nm (A260/280) was used to assess the purity of DNA and samples were diluted to 50 ng/µL with TlowE-buffer.

### Multiplex Polymerase Chain Reaction (Multiplex-PCR) for 16S rRNA Gene Amplicons

A barcoding-multiplex tandem PCR (Berry et al., 2012) was performed targeting the V3-V4 region of the bacterial 16S rRNA gene. Target and barcoding primers were designed as previously described (Hamady et al., 2008): HBact341**F**: 5’- CCTACGGGNGGCWGCAG-3’ and HBact785**R**: 5’- GACTACHVGGGTATCTA-3’.

The reaction mix contained 2 µL DNA (100 ng), 1x Taq buffer with KCl (B38), 0.2 mM dNTPs, 2 mM MgCl_2_, 1 µM forward and reverse primer, 0.1 µg/µL BSA and 25 mU/µL recombinant Taq Polymerase (all from Thermo Fisher Scientific). In the two-step PCR, the first round was performed in triplicates with final volumes of 20 µL per well and 25 cycles (95°C for 30 sec., 55°C for 30 sec. and 72°C for 60 sec.) and the second round was carried out with final volumes of 50 µL by addition of reaction mix (Taq buffer, dNTPs, MgCl_2_, BSA and Polymerase as described before) with 5 µL from first step pool, 2 µL barcoding primer, and 5 cycles (95°C for 30 sec., 52°C for 30 sec., and 72°C for 60 sec.). Amplicons were purified using the Zymo Research (ZR-96) sequencing DNA Clean-up Kit (D4017) and eluted in 20 µL per sample in PCR-grade water.

### Preparation and Sequencing

The amplification performance was checked by electrophoresis: each sample (5 µL with 1 µL 6X DNA Gel Loading Dye (Thermo Fisher Scientific)) was loaded on 120 mL 1.5% agarose gel (Biozym, LE Agarose) in 1x TBE (89 mM Tris, 89 mM boric acid, 2 mM EDTA) with 1.2 µL GelRed and compared to a ladder (Thermo Fisher Scientific, 1 kb DNA Ladder, ready-to-use). Electrophoreses was set up with 80 V (BIO RAD, PowerPac Basic Power Supply) for approx. 60 minutes and analysed with a Biorad, Molecular Imager Gel Doc XR+ System with Image Lab Software for bands approximating 500 base pairs considering an amplicon length of 513 base pairs was expected.

Amplicons were quantified using the Quant-i PicoGreen dsDNA Assay Kit (Thermo Fisher Scientific) by comparison to a standard curve measured with an Infinite M200 Microplate Reader (Tecan Trading AG with i-control™ software).

An equimolar pool of 2x10^10^ copies of amplicons per sample was prepared and sent to Mycrosynth AG (Balach, Switzerland) for sequencing on an Illumina MiSeq system.

### Bioinformatics

A total number of 2 840 051 sequences were aligned (Herbold et al., 2015) using MOTHUR (Schloss et al., 2009) and QIIME (Caporaso et al., 2010) by the Division of Microbial Ecology, University of Vienna with an expected amplicon length of 513 base pairs based on a paired end read. Unique sequences (singletons) were removed and remaining sequences were sorted according their unique 8 nt barcode. 1 348 195 merged read pairs were assigned to 749 operational taxonomic units (OTUs) at species-level, identified using a 97% identity threshold (Nguyen et al., 2016) and comparing to the SILVA database (Glockner et al., 2017).

Sequence data has been deposited in the NCBI Sequence Read Archive under SRP316524.

### UCB Measurements Using High-Performance Liquid Chromatography (HPLC)

Circulating UCB levels were measured in serum by HPLC following a well-established protocol (Wallner et al., 2013b; Seyed Khoei et al., 2020) using HPLC (HPLC, Merck, Hitachi, LaChrom, Vienna, Austria), equipped with a Fortis C18 HPLC-column (4.6 × 150 mm, 3 μm), a Phenomenex SecurityGuard™ cartridges for C18 HPLC-columns (4 × 3 mm), and a photodiode array detector (PDA, Shimadzu). An isocratic mobile phase contained glacial acetic acid (6.01 g/L) and 0.1 M n-dioctylamine in HPLC grade methanol/water (96.5/3.5%) was used. UCB was extracted from serum by mixing 40 μL serum with 160 μL mobile phase. After centrifugation, 50 μL of the supernatant was injected at a flow rate of 1 mL/min.

### *UGT1A1* Genotyping for TA Repeats in the UGT1A1*28 Promoter Region

*UGT1A1* alleles from whole blood were determined through melting curves, using QIAsymphony DSP DNA Midi Kits on a QIAsymphony SP automated system (QIAGEN). 10 μM working solutions of LightCyclerFastStart DNA Master HybProbe Mix and primers were run on a LightCycler 480 Instrument II (Roche), as described previously by von Ahsen et al. (2000).

### Statistical Analyses

Statistical analysis was performed using the computing environment R version 3.3.2 (R Development Core Team, 2017). Additional packages used were vegan (Oksanen et al., 2007) for alpha-, beta-diversity-analysis and Adonis/perMANOVA, cluster (Maechler et al., 2017) with wards method and indicspecies (Cáceres and Legendre, 2009) for indicator species analysis. Sequence data were subsampled for each sample to equal 95% of the counted sequences of the smallest sample size to avoid any bias from unequal sequencing depth. Generalized linear models were tested with the edgeR-package (McCarthy et al., 2012).

Normality was checked using the Shapiro-Wilk test in the coin-package of R (Hothorn et al., 2006). The non-parametric multivariate analyses of variance tests were performed by Adonis function which was set to 9999 permutations. Data are summarized according to their respective distribution. For parametric data, means ± SD (standard deviation), for non-parametric variables, medians ± IQR (inter-quartile range) are presented. For all statistical analyses, the significance level was based on P-value ≤ 0.05.

## Results

### Characteristics of the Study Population

Baseline characteristics of the study participants are presented in **Table 1**.

The GS group had significantly greater serum UCB levels and a reduced BMI (**Table 1A**), which was more pronounced in the older age group (above 35 years). This BMI difference between C and GS was more evident in females (**Table 1B**). The significant difference in UCB between groups was independent of sex (**Tables 1A–C**).

**Table 1:** Demographic description of the study subjects (all subjects/females/males).

A. Baseline characteristics of Gilbert’s Syndrome participants and their matched controls.			All subjects
	**C**	**GS**	**p-value**
Participants [n]	45	45	1.00
Sex (m/f)	29/16	29/16	
Median age [yrs]^Δ^	30.0 (19.0)	31.0 (18.5)	0.91
Participants aged ≤/> 35 yrs [n/n]	27/18	27/18	
Age of participants ≤/> 35 yrs [yrs]^Δ^	27.0 (6.0)/47.0 (12.25)	27.0 (6.0)/48.5 (13.5)	0.90/0.82
UCB concentration [µM]^Ø^	9.2 (3.4)	32.6 (9.4)	<0.001*
UGT1A1*28 TA repeats [7/6_7/6]^□^	3/21/21	40/4/1	
BMI [kg/m^2^]^Δ^	23.9 (6.2)	22.2 (3.4)	0.011*
BMI [kg/m^2^] ≤/> 35 yrs ^Δ^	22.8 (3.9)/27.2 (4.8)	21.5 (2.8)/22.8 (4.5)	0.135/0.015*
			
**B. Baseline characteristics of Gilbert’s Syndrome participants and their matched controls among females**.			**Female**
	**C**	**GS**	**p-value**
Participants [n]	16	16	1.000
Median age [yrs]^Δ^	40.0 (16.75)	40.5 (18.50)	0.949
Participants aged ≤/> 35 yrs [n/n]	7/9	7/9	1.000
Age of participants ≤/> 35 yrs [yrs]^Δ^	29 (4.0)/46 (13.0)	29.0 (3.5)/48 (12.0)	
UCB concentration [µM]^Ø^	8.6 (3.2)	30.1 (6.6)	<0.001*
UGT1A1*28 TA repeats [n 7/6_7/6]^□^	1/7/8	16/0/0	
BMI [kg/m^2^]^Δ^	23.9 (6.5)	20.6 (3.8)	0.024*
BMI [kg/m^2^] ≤/> 35 yrs^Ø^	21.0 (0.5)/27.5 (1.7)	21.4 (1.5)/20.7 (4.6)	0.549/0.012*
**C. Baseline characteristics of Gilbert’s Syndrome participants and their matched controls among males**.			**Male**
	**C**	**GS**	**p-value**
Participants [n]	29	29	1.000
Median age [yrs]^Δ^	29.0 (18.00)	29.0 (16.00)	0.944
Participants aged ≤/> 35 yrs [n/n]	20/9	20/9	1.000
Age of participants ≤/> 35 yrs [yrs]^Δ^	26 (6.0)/49 (10.0)	26 (5.5)/51 (12.0)	
UCB concentration [µM]^Ø^	9.9 (3.5)	33.9 (10.5)	<0.001*
UGT1A1*28 TA repeats [n 7/6_7/6]^□^	2/14/13	24/4/1	
BMI [kg/m^2^]^Δ^	23.9 (4.9)	22.4 (2.4)	0.101
BMI [kg/m^2^] ≤/> 35 yrs^Ø^	23.1 (4.4)/26.3 (7.0)	22.3 (2.7)/23.3 (3.6)	0.144/0.235

^Ø^Data are expressed as mean value ± SD. ^Δ^Data are expressed as median ± IQR.*p-values ≤0.05 show significant differences (t-test or Mann-Whitney-U-Statistic, respectively); Control (C) group and Gilbert’s syndrome (GS) group ^□^Insertion of additional TA repeats in the UGT1A1*28 promoter region; 7: Gilbert’s syndrome, 6_7: heterozygous individuals, 6: wild type.

### Composition of the Gut Microbiota

**Figure 1:**
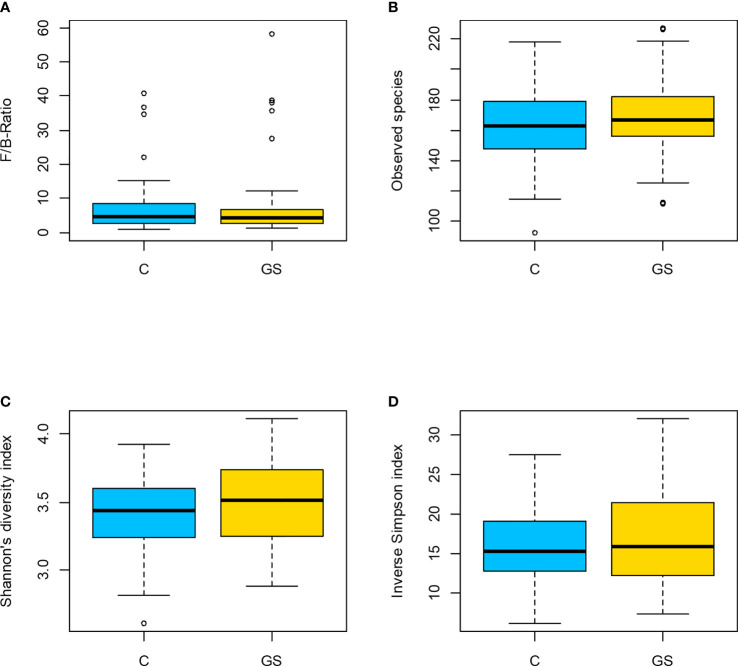
Plots of diversity characteristics: **(A)** Firmicutes to Bacteroidetes ratio, **(B)** observed species, **(C)** Shannon**’**s diversity index and **(D)** inv. Simpson index did not differ between the C/GS-groups (p > 0.05). In **(A)** participant B71 is not shown in C-group due to a F/B-ratio of 131.

**Figure 2:**
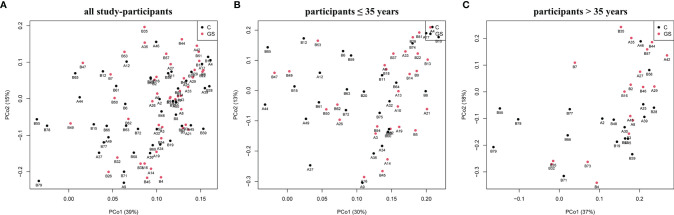
Plots of Principal coordinates analysis based on Bray-Curtis dissimilarity matrix for rarefied OTUs of C and GS samples and phenotype for **(A)** all study-participants and separated for age **(B)** ≤35 years and **(C)** >35 years. No distinct clusters are distinguishable.

Isolation and sequencing were successful with Good’s coverage between 98.5 – 99.7 % (**Table 2**) and rarefaction curves showed asymptotic behaviour, indicating that sufficient sequencing depth had been achieved. 749 OTUs were identified prior to rarefaction. The *Firmicutes*/*Bacteroidetes* ratio, alpha and beta diversity were not significantly different between C vs. GS-groups (**Table 2** and **Figure 1**), and PCoA ordination showed no distinct clusters (**Figure 2**).

**Table 2:** Medians sequencing data and diversity characteristics.

	C	GS	p-value
No. sequences	13793 (3682)	13647 (4821)	0.6
Good’s coverage	0.994 (0.0022)	0.994 (0.0020)	0.9
No. observed OTUs	159.6 (29.8)	163.4 (27.2)	0.2
Chao1	361.6 (78.2)	360.0 (87.8)	0.9
Shannon	3.438 (0.4)	3.520 (0.5)	0.2
inv. Simpson	15.28 (6.4)	15.771 (9.2)	0.3
F/B-ratio	4.52 (6.8)	4.25 (4.5)	0.5

Medians ± IQR are given. Mann-Whitney-U-statistic shows no significant difference between the control and Gilbert’s syndrome-group. Control (C) group and Gilbert’s syndrome (GS) group; OTU, Operational taxonomic units, F/B-ratio, Firmicutes/Bacteroidetes-ratio.

**Figure 3:**
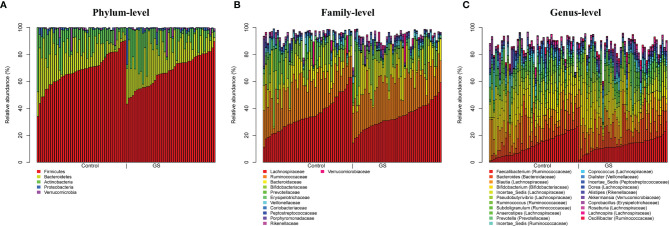
Plots of taxonomic profiles for **(A)** phylum-level, **(B)** family-level and **(C)** genus-level from BiliHealth-gut-samples for each group (Controls and Gilbert’s Syndrome). Abundant taxa with a mean relative abundance >1% are shown. There are no significant differences between C & GS group on all taxonomic levels.

Most abundant phyla (**Figure 3A**) in C and GS group were *Firmicutes* (C *vs.* GS: 68.4 ± 12.2% *vs.* 67.6 ± 11.5%, p ~ 0.92), *Bacteroidetes* (16.8 ± 10.8% *vs.* 18.4 ± 10.6%, p ~ 0.77), *Actinobacteria* (12.3 ± 8.9% *vs.* 10.9 ± 10.4%, p ~ 0.77), *Proteobacteria* (1.2 ± 1.7% *vs.* 1.8 ± 3.9%, p ~ 0.77), and *Verrucomicrobia* (1.1 ± 3.3% *vs.* 1.1± 3.4%, p ~ 0.99) (listed in decreasing order according C-group and abundance above 1%). At a family level (**Figure 3B****)** *Lachnospiraceae* (C *vs.* GS: 35.0 ± 12.4% *vs.* 34.0 ± 9.5%, p ~ 0.77), *Ruminococcaceae* (24.2 ± 8.3% *vs.* 25.6 ± 7.7%, p ~ 0.77), *Bifidobacteriaceae* (10.8 ± 8.7% *vs.* 9.5 ± 9.9%, p ~ 0.77), *Bacteroidaceae* (10.4 ± 8.2% *vs.* 12.4 ± 8.5%, p ~ 0.71), *Prevotellaceae* (3.2 ± 7.1% *vs.* 2.3 ± 6.6%, p ~ 0.77) and *Erysipelotrichaceae* (2.7 ± 3.0% *vs.* 1.6 ± 1.7%, p ~ 0.40), *Veillonellaceae* (2.0 ± 2.8% *vs.* 1.5 ± 1.8%, p ~ 0.72), *Rikenellaceae* (1.0 ± 0.9% *vs.* 1.5 ± 1.6%, p ~ 0.40), *Coriobacteriaceae* (1.6 ± 1.4% *vs.* 1.4 ± 1.1%, p ~ 0.77), *Peptostreptococcaceae* (1.5 ± 2.2% *vs.* 1.3 ± 1.4%, p ~ 0.72), *Porphyromonadaceae* (1.3 ± 3.1% *vs.* 1.3 ± 1.3%, p ~ 0.94), *Verrucomicrobiaceae* (1.1 ± 3.3% *vs.* 1.1 ± 3.3%, p ~ 0.99), *Streptococcaceae* (1.0 ± 1.4% *vs.* 1.0 ± 1.4%, p ~ 0.99), Enterobacteriaceae (0.7 ± 1.7% *vs.* 1.0 ± 4.2%, p ~ 0.77), *Christensenellaceae* (0.6 ± 09% *vs.* 1.0 ± 1.4%, p ~ 0.58) were predominant.

Abundances at a genus level (**Figure 3C**) were more diverse (listed in decreasing order according to C-group and abundance above 1%): *Faecalibacterium* (C *vs.* GC: 12.5 ± 7.6% *vs.* 12.8 ± 5.3%, p ~ 0.91), *Blautia* (11.3 ± 6.5% *vs.* 10.3 ± 4.0%, p ~ 0.71), *Bifidobacterium* (10.8 ± 8.7% *vs.* 9.5 ± 9.9%, p ~ 0.81), *Bacteroides* (10.4 ± 8.2% *vs.* 12.4 ± 8.5%, p ~ 0.69), Incertae_Sedis (*Lachnospiraceae*) (9.3 ± 4.1% *vs.* 8.1 ± 3.3%, p ~ 0.69), *Pseudobutyrivibri* (5.5 ± 4.1% *vs.* 6.7 ± 4.4%, p ~ 0.69), *Subdoligranulum* (3.3 ± 2.4% *vs.* 3.4 ± 2.8%, p ~ 0.92), *Prevotella* (3.0 ± 7.0% *vs.* 2.1 ± 6.6%, p ~ 0.81), *Anaerostipes* (3.1 ± 2.9% *vs.* 2.8 ± 2.4%, p ~ 0.84), *Ruminococcus (*3.0 ± 2.9% *vs.* 4.2 ± 3.3%, p ~ 0.69), Incertae_Sedis (*Ruminococcaceae*) (2.8 ± 3.5% *vs.* 2.0 ± 1.3%, p ~ 0.69), *Coprococcus* (1.7 ± 1.5% *vs.* 1.7 ± 1.6%, p ~ 0.96), *Dialister* (1.7 ± 2.7% *vs.* 1.3 ± 1.8%, p ~ 0.72), Incertae_Sedis (*Peptostreptococcaceae*) (1.5 ± 2.2% *vs.* 1.3 ± 1.4%, p ~ 0.85), *Dorea* (1.4 ± 0.9% *vs.* 1.3 ± 0.8%, p ~ 0.91), *Coprobacillus* (1.3 ± 1.8% *vs.* 0.9 ± 0.8%, p ~ 0.69), *Akkermansia* (1.1 ± 3.3% *vs.* 1.1 ± 3.3%, p ~ 1.00), Incertae_Sedis (*Erysipelotrichaceae*) (1.0 ± 2.2% *vs.* 0.6 ± 1.5%, p ~ 0.69), *Roseburia* (1.0 ± 1.0% *vs.* 1.1 ± 1.0%, p ~ 0.87), Collinsella (1.0 ± 1.2% *vs.* 0.9 ± 0.9%, p ~ 0.84), *Alistipes* (0.9 ± 0.9% *vs.* 1.5 ± 1.6%, p ~ 0.69), Streptococcus (0.9 ± 1.2% *vs.* 1.0 ± 1.4%, p ~ 0.91), *Lachnospira* (0.9 ± 1.15% *vs.* 1.1 ± 1.0%, p ~ 0.71), *Oscillibacter* (0.9 ± 1.0% *vs.* 1.1 ± 1.0%, p ~ 0.69) and unclassified (*Christensenellaceae*) (0.6 ± 0.9% *vs.* 1.0 ± 1.4%, p ~ 0.91).

### Gut Microbiota and Bilirubin Phenotype

Microbiota composition did not differ between the groups for all taxonomic levels and at OTU level based on the GS-phenotype and age (</≥ 35 years). The factor sex was a significant determinant of microbial composition at genus level with greater observed diversity in females (p < 0.05). Pairwise testing of the relative abundances of taxa on domain, phylum, class, family, genus and OTU-level showed no significant differences for GS-phenotype after adjusting for multiple testing with BH procedure (false discovery rate) with age, sex, and BMI as covariates using a generalized linear model.

An indicator species analysis (**Table 3**) resulted in very low abundance of OTUs and low association values (≤ 0.512) with a strong dependency to the rarefaction step performed during subsampling (described in *Statistical Analyses*). This low association values indicate that there is little association between OTU relative abundances and phenotype.

**Table 3:** Summary of an indicator species analysis.

	Assoc.	p-value	Closest related species	Accession	Identity [%]	C		GS	
						Mean abundance & sd [%]		Mean abundance & sd [%]	
C-group									
OTU_200	0.509	0.032*	*Emergencia timonensis strain SN18*	NR_144737.1	89	5.8E-02	8.6E-02	2.0E-02	3.3E-02
OTU_258	0.486	0.016*	*Acetanaerobacterium elongatum strain Z7*	NR_042930.1	90	1.9E-02	3.7E-02	1.1E-02	2.1E-02
OTU_230	0.394	0.015*	*Mitsuokella jalaludinii strain M9*	NR_028840.1	99	1.5E-01	5.2E-01	2.3E-03	7.4E-03
OTU_319	0.356	0.034*	*Olsenella scatoligenes strain SK9K4*	NR_134781.1	94	5.0E-02	1.6E-01	4.5E-03	1.6E-02
			* *						
GS-Group									
OTU_295	0.521	0.025*	*Christensenella minuta strain YIT 12065*	NR_112900.1	90	2.6E-02	5.6E-02	4.6E-02	5.9E-02
OTU_392	0.509	0.006**	*Prevotella intermedia strain B422*	NR_026119.1	99	1.3E-02	1.9E-02	2.0E-02	2.3E-02
OTU_465	0.464	0.01**	*Aggregatibacter aphrophilus strain CIP 70.73*	NR_116167.1	99	1.4E-02	1.9E-02	1.6E-02	2.0E-02
OTU_113	0.456	0.026*	*Rhodospirillum rubrum strain ATCC 11170*	NR_074249.1	87	2.0E-02	7.2E-02	2.4E-01	8.8E-01
OTU_109	0.438	0.022*	*Novispirillum itersonii strain NBRC 15648*	NR_113793.1	87	4.4E-02	2.5E-01	2.0E-01	5.7E-01
OTU_163	0.418	0.01**	*Kiloniella majae strain M56.1*	NR_152635.1	87	3.4E-03	8.8E-03	1.1E-01	3.9E-01
OTU_139	0.393	0.034*	*Bacteroides coprophilus strain CB42*	NR_041461.1	99	5.1E-03	2.7E-02	4.9E-01	2.1E+00
OTU_391	0.379	0.033*	*Spiroplasma alleghenense strain PLHS-1*	NR_025697.1	85	2.8E-03	9.8E-03	3.6E-02	9.9E-02
OTU_263	0.378	0.037*	*Ethanoligenens harbinense strain YUAN-3*	NR_074333.1	92	1.3E-02	4.2E-02	2.7E-02	7.6E-02
OTU_471	0.378	0.031*	*Parabacteroides distasonis strain ATCC 8503*	NR_074376.1	98	8.5E-03	4.3E-02	1.5E-02	4.6E-02

Table shows assignment of the best-hit result using BLAST to the given OTU-Sequence and their mean relative abundance in the Control (C) group and Gilbert’s syndrome (GS) group stratified by the result of the multipatt-function of the indicspecies-package. p-values were not adjusted for multiple-testing but tested by permutation option: control = how(nperm=999) *p-values ≤ 0.05 and **p-values ≤ 0.01.

## Discussion

Mild hyperbilirubinaemia (GS) with normal circulating liver transaminases, biliary markers, and red blood cell counts, is a benign condition that is highly prevalent among the general population. Elevated UCB levels are inversely associated with the risk of chronic diseases including some cancers. Since bilirubin is in part metabolised in the gut, we investigated whether participants with mildly increased UCB levels exhibit a different gut microbiota composition compared to age- and sex-matched controls. Such differences could help to better explain the link between lower CRC risk observed in GS individuals, but could also be linked to the lower risk for metabolic diseases.

In the present study, gut microbiota composition was determined using 16S rRNA gene-targeted sequencing, a popular approach to determine whether there are alterations in the microbiota linked to disease states (Rebolledo et al., 2017; Leiva-Gea et al., 2018; Das et al., 2021). Microbial patterns that are typically associated with proximal or distal CRC could not be detected in either group. Compared to controls no differences between alpha and beta diversity and no over- and underrepresentation of genera were detected in GS individuals. The bacterial community in both groups was dominated by the typical phyla *Firmicutes* and *Bacteroidetes*. A difference in the percentage of *Firmicutes* and *Proteobacteria*, which have been associated with the mucosa of CRC patients (Gao et al., 2015) was not detected. Similarly, no difference was found in the ranks of family and genus. While the lack of a statistically significant association between the microbiota and GS could be due to cohort size, similar-sized studies have found differences in the microbiota within disease states (Rebolledo et al., 2017; Leiva-Gea et al., 2018; Das et al., 2021; Shuntian et al., 2021). As this is a reasonable cohort size for a pilot study, we conclude that if the microbiota is affected by GS, it must be a relatively minor effect on community composition.

In the literature, only four bacteria (*Bacteroides fragilis, Clostridium ramosum, Clostridium perfringens*, and *Clostridioides difficile*) have been linked to bilirubin metabolism so far, since they were able to reduce urobilinogen mixtures, including half-stercobilinogen and stercobilin, under *in vitro* and *in vivo* conditions (Chen and Yuan, 2020). These species are all common members of the intestinal microbiota (Vitek et al., 2005; Hamoud et al., 2018).

Although microbiota data of GS have not been published previously, there are a small number of reports from newborns with jaundice, which experience much higher and likely pathogenic bilirubin concentrations in the blood, when compared to GS. In jaundiced neonates, *Clostridium perfringens* was significantly elevated, which was considered as feedback on the severe hyperbilirubinemic conditions in the neonates (Dong et al., 2018). Further, *in vitro* data show that bilirubin is protective to the bacterial pathogen *Escherichia coli O157:H7*, but highly toxic to the bacterium *Enteroccocus faecalis* (Nobles et al., 2013).

Due to non-robust results of our OTU analysis, we could not reliably identify indicator taxa. Intriguingly, no OTUs classified to the above-mentioned bacteria were identified as indicators for GS in this cohort. Regardless of the latter, OTU_295 with a sequence identity of 90% (366/407 nucleotides with 4 gaps) was identified with the highest association value (0.521) that can be assigned to the GS-group. A subsequent BLAST (Zhang et al., 2000) analysis identified *Christensenella minuta* strain YIT 12065 (Morotomi et al., 2012) as the species closest to the sequence of OTU_295. Indeed, Goodrich et al. (2014) had previously associated this species with lean body mass. As GS individuals have a comparably lower BMI (Seyed Khoei et al., 2018) this result could be a first link to a lower CRC and potentially CVD risk.

Both study groups were generally very healthy, which could have masked potential microbial patterns. Sex was the only factor contributing to a slight difference in the composition of the genus-level microbiota profile, which has also been reported in other studies (Kim et al., 2020) but the lack of other associations could also be due to confounding factors in our cohort such as diet, race, medications or BMI.

Bile plays an important part in bilirubin metabolism. Upon conjugation, multidrug resistance-associated protein 2 transports conjugated bilirubin into the duodenum *via* the biliary tract and passes through the small intestines until it reaches the distal ileum and colon. Conjugated bilirubin is then again deconjugated by bacterial β-glucuronidases. Mainly in the large intestine, the intestinal microbiota metabolise UCB to urobilin and stercobilin. However, part of the deconjugated bilirubin of the bile is reabsorbed as part of the enterohepatic circulation before reaching the rectum (Bulmer et al., 2011; Chen and Yuan, 2020). Bile acids in bile are also secreted in the intestinal lumen and are subsequently re-absorbed in the terminal ileum and transported back to the liver for recycling. Some bile acids, however, reach the colon and are modified by the gut microbiota, which affects their physicochemical properties as well as inhibitory activity on bacteria. Therefore, bile acids also shape the composition and function of the intestinal microbiota. While there is no data available about a potential differences in bile acid composition within GS subjects, data from a genome wide analysis show that the *UGT1A1* GS SNP variant rs6742078 is associated with gallstone disease in men (Buch et al., 2010), which might also affect gut microbiota composition. More data are needed in future to explore this question and to better understand the interplay between bile acids, bile pigments and microbiota composition.

We were not able to analyse UCB and stercobilin in the faeces of the subjects. Concentrations of both metabolites should be complementary, considering previous experiments demonstrating an increase in DNA strand breaks in human cancer cells depending on the concentration of these bile pigments (Mölzer et al., 2013b). Further, mutagenesis induced by the food-borne mutagen aflatoxin B1 was abrogated by both urobilin and stercobilin in the AMES Test. These findings point towards the importance of these compounds in gut metabolism and the interplay with food derived mutagens, which play a role in CRC development (Mölzer et al., 2013a).

In conclusion, this study indicates that, in the absence of acute inflammation or neoplasia, mildly elevated chronic UCB concentration in the blood in GS, which is associated with improved metabolic health, is not associated with an altered gut microbial composition when compared to a healthy age- and sex- matched control group.

## Data Availability Statement

Sequence data has been deposited in the NCBI Sequence Read Archive under SRP316524.

## Ethics Statement

The studies involving human participants were reviewed and approved by Ethics Commission of the Medical University of Vienna. The patients/participants provided their written informed consent to participate in this study.

## Author Contributions

K-HW, CM, and MH-W designed the study and collected data. PZ, CM, and MH-W performed the analyses. PZ and K-HW drafted the manuscript with input from all authors. PZ performed formal analyses and provided all figures and tables. All co-authors supported in interpreting the results. K-HW and DB supervised the project covering different fields of scientific expertise. All authors contributed to the article and approved the submitted version.

## Funding

PZ was funded by the FWF Stand-Alone Project P 29608 and supported by the Vienna Doctoral School of Pharmaceutical, Nutritional and Sport Sciences’ Completion Grant of the University of Vienna.

## Conflict of Interest

The authors declare that the research was conducted in the absence of any commercial or financial relationships that could be construed as a potential conflict of interest.

## Publisher’s Note

All claims expressed in this article are solely those of the authors and do not necessarily represent those of their affiliated organizations, or those of the publisher, the editors and the reviewers. Any product that may be evaluated in this article, or claim that may be made by its manufacturer, is not guaranteed or endorsed by the publisher.
